# Focus on tricuspid valve—the European perspective

**DOI:** 10.1093/eurheartjsupp/suaf099

**Published:** 2026-03-14

**Authors:** Helge Möllmann, Marcel Weber, Maria Isabel Körber, Theresa Ann-Maria Gößler, Tobias Friedrich Ruf, Hannah Kempton, Paolo Denti, Rodrigo Estevez-Loureiro, Christina Grothusen, Volker Rudolph, Jörg Hausleiter, Holger Thiele

**Affiliations:** Medizinische Klinik I, St. Johannes Hospital Dortmund, Dortmund, Germany; Medizinische Klinik und Poliklinik II, Universitätsklinik Bonn, Bonn, Germany; Klinik III für Innere Medizin, Universitätsklinik Köln, Cologne, Germany; Zentrum für Kardiologie, Universitätsmedizin Mainz, Mainz, Germany; Zentrum für Kardiologie, Universitätsmedizin Mainz, Mainz, Germany; Medizinische Klinik und Poliklinik I, Ludwigs-Maximilians Universität München, Munich, Germany; San Raffaele University Hospital, Milan, Italy; Department of Interventional Cardiology, Hospital Alvaro Cunqueiro, Vigo, Pontevedra, Spain; Medizinische Klinik I, St. Johannes Hospital Dortmund, Dortmund, Germany; Klinik für Herzchirurgie, UKSH Kiel, Kiel, Germany; Allgemeine und interventionellen Kardiologie, HDZ NRW Bad Oeynhausen, Bad Oeynhausen, Germany; Medizinische Klinik und Poliklinik I, Ludwigs-Maximilians Universität München, Munich, Germany; Herzzentrum Leipzig, Leipzig, Germany

**Keywords:** Tricuspid regurgitation, Interventional treatment, TEER, Valve implantation, Heart failure

## Abstract

Tricuspid regurgitation (TR) affects approximately 4% of individuals over 75 years of age and is associated with substantial morbidity due to heart failure symptoms and frequent hospitalization. In Europe, TR prevalence is expected to rise with an ageing population, contributing to a growing burden on heart failure services. Surgical repair or replacement for isolated TR has been historically underutilized because of high operative risk, however, recent advancements in transcatheter technology have shifted the treatment paradigm. Having once been labelled the forgotten valve, the European interventional cardiology community was suddenly confronted with a number of different devices, all designed to target TR. Having the intrahospital mortality for isolated tricuspid valve surgery in mind, ranging from 8.0% to 12.3%, the initial results of transcatheter therapies with an all-cause mortality of 3.7% at 30 days were promising, although procedural success was achieved in only 62% and cardiac and cerebrovascular major adverse events were as high as 26%. Already, T-TEER constituted the majority of interventions, although miskeyed M-TEER devices were used off-label. Other systems, such as Trialign, TriCinch, FORMA, Cardioband, NaviGate, and caval valve implantation were used distinctively less often.

## Introduction

Tricuspid regurgitation (TR) affects approximately 4% of individuals over 75 years of age and is associated with substantial morbidity due to heart failure symptoms and frequent hospitalization.^1,2^ In Europe, TR prevalence is expected to rise with an ageing population, contributing to a growing burden on heart failure services.^3^ Surgical repair or replacement for isolated TR has been historically underutilized because of high operative risk; however, recent advancements in transcatheter technology have shifted the treatment paradigm.^4^

**Table 1.** 

Author	Title	Devices	Patients (n)	Age	Female	Comorbidities	FUP(m)	Outcomes assessed	Year
	**TRIVALVE-Registry**	Several							
Taramasso *et al*.^5^	The International Multicenter TriValve Registry Which Patients Are Undergoing Transcatheter Tricuspid Repair?	MitraClip (*n* = 58), Trialign (*n* = 17), TriCinch (*n* = 15), FORMA (*n* = 7), Cardioband (*n* = 5), and caval valve implantation (*n* = 3).	106	76 ± 9	60.4%	EuroSCORE II 7.6 ± 5.7%; 95% NYHA III or IV	1	Early experience with wide array of TTVI in high risk patient population with mostly STR. Procedural success at 62%, TTVI alone in 68%. AT 30 days, all-cause mortality was 3.7%, MACE of 26%, and NYHA-fc I or II 58%.	2017
Taramasso *et al*.^5^	Outcomes After Current Transcatheter Tricuspid Valve Intervention Mid-Term Results From the International TriValve Registry	MitraClip (210), Trialign (18), TriCinch first generation (14), caval valve implantation (30), FORMA (24), Cardioband (13), NaviGate (6), and PASCAL (1)	312	76.4 ± 8.5	57%	ES II 9 ± 8	18	‘Mid-Term results’—procedural success 72.8%, 30-day mortality 3.6% and lower in those with procedural success (1.9% vs. 6.9%). Tethering (=greater coaptation depth) identified as independent predictor for device failure. Survival at 18 months (1.5 years) 82.8 ± 4%.	2019
Mehr *et al*.^27^	1-Year Outcomes After Edge-to-Edge Valve Repair for Symptomatic Tricuspid Regurgitation Results From the TriValve Registry	T-TEER Only	249	77 ± 9		ES II 6.4% [3.9%; 13.9%] ; NYHA III or IV 95.6% (NYHA taken from Karam, below)	12	Focus on T-TEER, with acute procedural success as residual TR ≤2+ in 77% at 30dFUP and 72% at 1yFUP. All-cause 1-year mortality 20% with the predictors being worsening kidney function, non-sinus rhythm and procedural failure. Predictors for procedural failure large EROA, Gap, Tenting area, and non-S/A jet location.	2019
Taramasso *et al*.^5^	Transcatheter Versus Medical Treatment of Patients With Symptomatic Severe Tricuspid Regurgitation	TTVI vs. OMT	268 matched pairs (472 vs. 1,179)				12	Matching of the TRIVALVE registry (*n* = 472) to 2 retrospective registries (*n* = 1.179) in Europe and North America. Lower mortality (23 ± 3% vs. 36 ± 3%) and hospitalizations for TTVI (unadjusted HR 0.60; 95% CI: 0.46–0.79; adjusted HR: 0.35; 95% CI: 0.23–0.54).	2019
Mehr *et al*.^27^	Combined Tricuspid and Mitral Versus Isolated Mitral Valve Repair for Severe MR and TR An Analysis From the TriValve and TRAMI Registries	TEER Only	228 (122 TRIVALVE vs. 106 TRAMI)	77 ± 8	44.3%	NYHA III or IV 93.9%	12	Comparison of TMVI only (TRAMI) vs. TMVI+TTVI (TRIVALVE) in patients with severe MR and TR. After multivariable analysis, NYHA-fc did not differ but 1y-mortality was lower for TMVI+TTVI (16.4% vs. 34.0%, HR 0.52).	2020
Karam *et al*.	Value of Echocardiographic Right Ventricular and Pulmonary Pressure Assessment in Predicting Transcatheter Tricuspid Repair Outcome	T-TEER Only	249			ES II 6.4% [3.9%; 13.9%] (taken from Mehr, above); NYHA III or IV 95.6%	12	Echocardiographic assessment of RV function and pulmonary pressure did not predict outcome for T-TEER.	2020
Coisne *et al*.^28^	Prognostic Value of Tricuspid Valve Gradient After Transcatheter Edge-to-Edge Repair: Insights From the TriValve Registry.	T-TEER Only	308				12	Baseline Tricuspid Valve gradients do not influence the outcome after T-TEER.	2023
Rommel *et al*.^29^	Low-Cardiac Output Syndrome After Tricuspid Valve Repair Insights From the TriValve Registry	Several	500	78 [73; 82]		ES II 6% [4; 12]	only in-hospital	Low CO following TTVI predicted by impaired baseline RVPAC (*P* = 0.05), biventricular impairment (*P* < 0.01), procedural complications (*P* < 0.01), pre-procedural hemodynamic instability (*P* < 0.01), and EuroSCORE II >15% (*P* < 0.01).	2023
Russo *et al*.^17^	Characteristics and outcomes of patients with atrial versus ventricular secondary tricuspid regurgitation undergoing tricuspid transcatheter edge-to-edge repair—Results from the TriValve registry	T-TEER Only	298				12	aSTR and vSTR are recognized as distinct pathologies with T-TEER achieving similar results in procedural success and effectiveness in both, but vSTR suffering from lower survival (72% vs. 91%; vSTR with HR 4.75 as independent predictor for mortality).	2023
Russo *et al*.^17^	Effects of tricuspid transcatheter edge-to-edge repair on tricuspid annulus diameter—Data from the TriValve registry	T-TEER Only	186					T-TEER shows an acute indirect annuloplasty effect in 62% of patients, but not when ≥3 implants are used.	2024
Cannata *et al*.^30^	Mitral regurgitation evolution after transcatheter tricuspid valve interventions-a sub-analysis of the TriValve registry	Mostly T-TEER (80%)	359				2	MR is regularly affected following TTVI, and most often reduced. Predictors for MR improvement were AFib, T-TEER, procedural success, TR reduction, LVEDD > 60 mm, and beta-blocker therapy, while annuloplasty and heterotopic TTVR were associated with MR worsening. Patients with MR ≥ moderate-to-severe following TTVI showed significantly higher death rates.	2024
Pancaldi *et al*.	Right heart failure and mortality in patients undergoing transcatheter tricuspid valve interventions	Several	498 (of 639)				12	RHF at baseline is common among patients undergoing TTVI and has a negative impact on mortality when more than 1 criterion (prev. hospitalization for RHF, signs—jugular venous distension, ascites, peripheral oedema—, ≥ 125 mg/d furosemide or equivalent) is present (HR 2.91–95%; CI 1.46–5.83).	2025
	**EURO-TR-Registry**	T-TEER only							
Stolz *et al*.^15^	Residual tricuspid regurgitation after tricuspid transcatheter edge-to-edge repair: Insights into the EuroTR registry		1286	78.0 ± 8.9	53.6%		24	T-TEER is feasible, safe, and effective with durable results at 2 yFUP. Residual TR ≧ 3/5 was an independent predictor of all-cause mortality (HR 2.06, 95% CI 1.30–3.26), while there was no difference in patients with residual TR 1/5 vs. 2/5. Residual high TR was associated with higher baseline TR and with higher tenting area. Symptom reduction was associated with degrees of TR reduction.	2024
Kassar *et al*.^24^	The prognostic value of the Dandel's index in patients undergoing tricuspid transcatheter edge-to-edge repair		364			NYHA III or IV 92%	24	Mortality among patients with severe TR is high (36% after 2 years); Dandel's index to adjust for R loading conditions predicted all-cause mortality and hospital readmission for heart failure (Threshold 20.5).	2024
Stolz *et al*.^15^	Atrial Secondary Tricuspid Regurgitation Insights Into the EuroTR Registry		641	79 ± 7	50		24	Prevalence of aSTR is 31%, with association to more AFib and less comorbidities than vSTR. T-TEER in aSTR might be more effective. Survival in patients with aSTR was higher.	2024
Schlotter *et al*.^20^	Tricuspid Regurgitation Disease Stages and Treatment Outcomes After Transcatheter Tricuspid Valve Repair		1885				12	Introduction of disease stages. Comparison of T-TEER vs. conservative treatment showed no mortality difference in the early and advanced stages. Mortality benefit for patients in the intermediate group treated with T-TEER (HR 0.73; 95% CI 0.52–0.99). Underlines the importance of patient selection, including when to treat.	2025
Stolz *et al*.^25^	Impact of Pulmonary Hypertension on Outcomes After Transcatheter Tricuspid Valve Edge-to-Edge Repair		1230	78.6 ± 7.0	51.4		24	Impact of PHT on T-TEER patients: Increasing sPAP is associated with increasing mortality or HFH. No outcome differences between pre- vs. post-capillary PHT.	2025
Pagnesi *et al*.^23^	Malnutrition and outcomes in patients with tricuspid regurgitation undergoing transcatheter tricuspid valve repair		1034	78.4 ± 7.3 years	52.3		24	Malnutrition, as defined by the Geriatric nutritional Risk Index (GNRI) ≤ 98, is common with a prevalence of 20.4%. It is associated with increased all-cause mortality (HR 1.53, 95% CI 1.11–2.10). Residual higher TR is associated with a worse outcome, irrespective of GNRI.	2025

	**PASTE-Registry**	T-TEER only							
Wild *et al*.^26^	Multicenter Experience With the Transcatheter Leaflet Repair System for Symptomatic Tricuspid Regurgitation		235	78 ± 8	49	STS 8.6% ± 6.8%	6	Early feasibility and efficacy of Pascal for T-TEER, showing reduction of TR to ≤2/5 in 78% of patients, along with improvements in NYHA-FC to I/II in 63%. There was no difference in between devices P10 vs. P5.	2022
Wild *et al*.^26^	Transcatheter Valve Repair for Tricuspid Regurgitation 1-Year Results From a Large European Real-World Registry		1059	79 ± 9	53	TRI-SCORE risk 23% ± 18; NYHA-FC III/IV 87%	12	1-year data of Pascal for T-TEER with similar feasibility and efficacy. Independent predictors for residual TR > 2/5: CGS > 8 mm, tenting height ≥ 10 mm, transvalvular lead, RV-dilatation > 42 mm, baseline TR ≥ 4/5. Centre experience (≥21 patients/a) and Pascal P5 each associated with better outcomes.	2025
	**TRIGISTRY**	Several							
Dreyfus *et al*.^4^	TRI-SCORE: a new risk score for in-hospital mortality prediction after isolated tricuspid vale surgery	ITVS	466	60 ± 16 years	49	nn	only in-hospital	Implementation of the TRI-SCORE.	2022
Dreyfus *et al*.^22^	TRI-SCORE and benefit of intervention in patients with severe tricuspid regurgitation	GDMT, ITVS, T-TEER	2413	Nn	Nn	TRI-SCORE was low (≤3) in 32%, intermediate (4–5) in 33%, and high (≥6) in 35%.	24	Survival progressively decreased with the TRI-SCORE irrespective of treatment modality.	2024
Adamo *et al*.^3^	Prediction of Mortality and Heart Failure Hospitalization After Transcatheter Tricuspid Valve Interventions Validation of TRISCORE	Several	634	Nn	Nn	35.2%) had a TRI-SCORE between 0 and 5, 221 (34.8%) had 6 or 7, and 190 (30%) had ≥8 points		In the TriValve registry, the TRI-SCORE has a suboptimal performance in predicting clinical outcomes.	2024
Dreyfus *et al*.^22^	Prognostic Implications of Residual Tricuspid Regurgitation Grading After Transcatheter Tricuspid Valve Repair	Only TEER	613	Nn	Nn	Nn	24	Importance of achieving a mild to moderate or lower residual TR grade during TTVI is highlighted.	2024
Dreyfus *et al*.^22^	Benefit of isolated surgical valve repair or replacement for functional tricuspid regurgitation and long-term outcomes stratified by the TRI-SCORE	ITVS vs. GDMT	1768 (551 surgery), 1217 conservatively)	Nn	Nn	TRI-SCORE distribution was 33% low, 32% intermediate, and 35% high	120	Higher survival rates were observed with repair than replacement and benefit of intervention declined as TRI-SCORE increased with no benefit of any type of surgery in the high TRI-SCORE category.	2024

	**TRICLASP**	T-TEER (Pascal)							
Baldus *et al*.^12^	Transcatheter valve repair of tricuspid regurgitation with the PASCAL system: TriCLASP study 30-day results		74	80.3	58.1%	STS-Score (MVr) 9%; NYHA III/IV in 77%	1	T-TEER with Pascal is feasible, effective with 90% sustaining TR ≤ 2/5 and NYHA I/II of 55.8% and experiencing increased 6MWD (+38.2 m) and KCCQScore (+13.4) at 1mFUP. Mortality was 1.5% and MAE-rate 3%.	2022
	**bRIGHT**	T-TEER with TriClip							
Lurz *et al*.^9^	Short-Term Outcomes of Tricuspid Edge-to-Edge Repair in Clinical Practice			79 ± 7 years	Nn	80% NYHA III or IV	1	Transcatheter tricuspid valve repair was found to be safe and effective in treating significant TR in a diverse, real-world population.	2023
Donal *et al*.^18^	Characterization of Tricuspid Valve Anatomy and Coaptation Gap in Subjects Receiving Tricuspid Transcatheter Edge-To-Edge Repair: Observations From the bRIGHT TriClip Study	T-TEER with TriClip	135	Nn	Nn	nn	1	Thirty-day TR reduction (by number of grades) was similar among subjects with coaptation gaps of <7 mm, 7–10 mm, and >10 mm.	2024
Lurz *et al*.^14^	Real-World 1-Year Results of Tricuspid Edge-to-Edge Repair From the bRIGHT Study	T-TEER with TriClip	511	79 ± 7 years	Nn	80% NYHA III or IV	12	Significant improvements in NYHA functional class (21–75% I/II, *P* < 0.0001) and Kansas City Cardiomyopathy Questionnaire (KCCQ) score (19 to 26-point improvement, *P* < 0.0001) were observed at 1 year. One-year mortality was lower in subjects who achieved moderate or lower TR at 30 days	2024
Goebel *et al*.	Outcomes of tricuspid transcatheter edge-to-edge repair in subjects with endocardial leads	T-TEER with TriClip in in subjects with endocardial leads	110	Nn	Nn	Nn	1	In the bRIGHT EU PAS, T-TEER using the TriClip system was safe and effective in severe TR subjects with an endocardial lead across the TV.	2025
	**European EVOQUE Registry**	TTVR (EVOQUE)							
Angelolotti *et al*.	Early Outcomes of Real-World Transcatheter Tricuspid Valve Replacement		176	77.8	72%	TRI-SCORE 5	1	T-VARC clinical success 86.9%, NYHA-FC I/II 71%, mortality 5.1%, PPM-implantation in 18.9% (PPM-naive).	

Having once been labelled the *forgotten valve*, the European interventional cardiology community was suddenly confronted with a number of different devices, all designed to target TR. Having the intrahospital mortality for isolated tricuspid valve surgery in mind, ranging from 8.0% to 12.3%, the initial results of transcatheter therapies with an all-cause mortality of 3.7% at 30 days were promising, although procedural success was achieved in only 62% and cardiac and cerebrovascular major adverse events were as high as 26%. Already, T-TEER constituted the majority of interventions, although mis-keyed M-TEER devices were used off-label. Other systems, such as Trialign, TriCinch, FORMA, Cardioband, NaviGate, and caval valve implantation were used distinctively less often.^5^

## Development of a new therapeutic target

The first report of an off-label tricuspid transcatheter edge-to-edge repair (T-TEER) with a MitraClip (Abbott, Abbott Park, IL, USA) system in a patient undergoing simultaneous mitral transcatheter edge-to-edge repair (M-TEER) was published in 2015.^6^ In this case, the steerable guide was positioned with a perpendicular trajectory in relation to the tricuspid plane and the device delivery catheter was inserted as conventionally done for M-TEER. This was later modified in a way that the guide catheter was pointing anteriorly, away from the septum, and an anterior-posterior orientation of the flex curve of the device delivery catheter by a 90°-offset insertion compared to the conventional mitral orientation. The addition of the S/L knob for the specific TriClip-system ((Abbott, Abbott Park, IL, USA) was introduced with the G3 system and moved the main steering manoeuvre from the guide delivery system to the steerable guide and allowed to direct the flex point of the device delivery catheter away from the septum and to avoid ‘septal hugging’. At this stage, two device sizes (NT and XT) were available. It received CE-mark in 2019. In 2021 G4 with 4 different device sizes and different widths (NT, NTW, XT, and XTW) was CE-marked and currently the G5 system has entered the market.

Early reports of tricuspid TEER (T-TEER) with the MitraClip device described reductions of TR by at least one grade in ≥ 91% of patients with already low complication rates.^7^ In 2017, the re-designed Triclip system with a modified short-tipped guide catheter, which was used in combination with the third-generation NT clip device was introduced and investigated in the TRILUMINATE EFS trial. Reduction of TR grade ≤2 + was achieved in 56% of patients.^8^ In 2019, the fourth generation of clip devices, now with four different sizes and independent leaflet grasping, was released. Reduction of TR to grade ≤2 + was reached in 80% of patients.^9^ The TRILUMINATE pivotal trial, which used the fourth-generation device in 70.1% of cases was initiated in 2019.^10^

The PASCAL repair system is the other currently approved T-TEER device. The first implant type, PASCAL, was released with a paddle width of 10 mm and approved for M-TEER in 2019. In 2020, the PASCAL Ace system with a narrower paddle width of 6 mm was added and the PASCAL system was approved for T-TEER in Europe. Interestingly, the same delivery unkeyed system, which allows manoeuvres in three planes, is used for mitral and tricuspid TEER. The PASCAL system was the first system to incorporate independent leaflet grasping which is used in most T-TEER procedures. Another interesting feature of these implants is the ability to elongate the system, which allows to easily avoid chordal interactions in a safe manner. The CLASP TR feasibility trial showed TR reductions to grade 2 + or less in 52% of cases.^11^ In the European post-market TriCLASP registry this device reached TR grade ≤ 2 + in 90% of patients.^12^

Overall, there is a rapid increase in the adoption of T-TEER worldwide, with large variations depending on the reimbursement situation and more than 26 000 procedures have been performed to date. In Germany, T-TEER increased from a total of 74 in 2012 to 2015 to 4298 cases in 2023 indicating an exponential rise.^13^

## European perspective—registry derived data

### Patient characteristics

Patients undergoing transcatheter treatment for TR in Europe have been characterized in several multicentre registries, including TRIVALVE, EuroTR, and bRIGHT. These datasets provide insights into the demographic and clinical profiles of patients treated in routine clinical practice across diverse European healthcare settings.

### Patient demographics

Across these cohorts, patients were elderly, with mean ages ranging from 76 to 79 years, and a female predominance of approximately 54–60%. bRIGHT reported a mean age of 79 ± 7 years and 56% female patients, with similar findings in EuroTR and TRIVALVE.^5,14,15^

Most patients were highly symptomatic, with 80–95% classified as New York Heart Association functional class (NYHA-fc) III or IV at baseline. A substantial proportion presented with highest grades of TR, specifically 50.7% exhibiting massive or torrential TR in TRIVALVE and up to 88% in the bRIGHT cohort.

### Echocardiographic assessments

Right ventricular (RV) dysfunction was frequently observed, as evidenced by tricuspid annular plane systolic excursion (TAPSE) < 17 mm in 46.2% of patients in EuroTR and 56.3% in TRIVALVE. This was often accompanied by elevated systolic pulmonary artery pressures, reflecting a high prevalence of pulmonary hypertension within this population.^5^

TR was predominantly secondary in nature, accounting for approximately 85–95% of cases, with primary and mixed aetiologies comprising the remainder. Among secondary TR cases, atrial secondary TR (aSTR) represented around 20–30%, characterized by a higher incidence of atrial fibrillation, fewer comorbidities, better biventricular function, less leaflet tenting, and enlarged atria. The majority of ventricular secondary TR (vSTR) was associated with higher incidence of left heart disease as a possible cause.^16^

Echocardiographic baseline assessments in patients treated for severe TR with transcatheter tricuspid valvular interventions (TTVI) consistently demonstrated significant anatomical alterations. The mean tricuspid annulus diameter was similarly dilated across registries, measuring 45.4 ± 11 mm in TRIVALVE and 45.2 ± 8.4 mm in EuroTR, resulting in a predominantly central regurgitant jet.

Coaptation gap size (CGS)—a critical parameter for procedural planning—averaged 6.3 ± 3.1 mm in EuroTR and 6.49 ± 2.7 mm in bRIGHT. More detailed echocardiographic analyses from bRIGHT using transgastric short-axis views demonstrated regional differences, with coaptation gaps of 8.1 ± 3.1 mm and 5.2 ± 2.3 mm in the central and mid anterior-septal coaptation line, and 6.6 ± 3.2 mm and 3.8 ± 2.1 mm in the central and mid septal-posterior coaptation line, respectively.^17,18^

### Pacemaker leads

A significant subset of patients undergoing TTVI had prior transvenous device leads implanted, present in approximately 23–28% of cases. Importantly, in about 80.7% of these patients, TR was at least partially attributed to their presence, underscoring the significant role of device-associated TR in this population.^14,15^

### Disease stages and its influence on outcome

As in any interventional treatment, patient selection is key to success. Suffering from high morbidity; this specifically applies to patients treated for TR using TTVI. For T-TEER, Schlotter *et al*. devised a disease grading scheme based on biventricular and kidney function, as well as NT-proBNP levels, that demonstrates that treating too late will not change the dismal course of TR.^19^

### Risk scores and prognostic markers in tricuspid regurgitation

Accurate risk stratification is essential for optimal patient management, leading to the development of targeted prognostic scores. Notably, the TRI-SCORE and the TRIVALVE score were conducted within European registries, next to further important clinical and echocardiographic markers.^4,20^

An important development was the TRI-SCORE, a risk model specifically created to predict in-hospital mortality after isolated tricuspid valve surgery comprising echocardiographic parameters like right and left ventricular function as well as age, signs of right heart failure and renal function. Validated in the extensive TRIGISTRY registry, TRI-SCORE outperformed general cardiac surgery risk scores in TR patients and demonstrated that patients with low scores (≤3 points) experience significantly better outcomes when treated surgically or via transcatheter methods compared to conservative therapy, whereas patients with high scores (≥6 points) show limited mortality benefit from intervention (2-year survival ∼60% in all groups).^21^

These findings underscore that advanced disease stage attenuates the benefits of intervention and highlight the importance of early risk assessment. Similarly, the TRIVALVE score emerged from the TriValve registry to predict outcomes after TTVI. It considers atrial fibrillation, renal function, liver congestion markers, clinical RHF, and left ventricular dysfunction. Patients with TRIVALVE scores above 2.5 are identified as higher risk for adverse outcome like heart failure hospitalization and mortality. These scores help to guide patient selection and procedural planning effectively.

Clinical markers further refining prognosis include RHF symptoms, nutritional status, RV functional indices, and pulmonary hypertension (PH). The EuroTR registry underscored malnutrition as an independent adverse prognostic factor, significantly increasing mortality risk post-intervention. Importantly, successful TR reduction consistently improved outcomes across nutritional statuses, emphasizing early nutritional assessment and intervention as integral to management.^22^

RV function remains pivotal in TR prognosis. The EuroTR-derived analysis of Dandel’s index, a load-adjusted echocardiographic RV function measure, identified reduced RV adaptability as independently associated with higher mortality and hospitalization risk. This metric surpasses conventional measures like TAPSE in predicting outcomes, indicating that detailed RV assessment should guide intervention timing.^23^ Pulmonary hypertension as another critical prognostic marker investigated in the EuroTR registry, is prevalent among TR patients. An increased systolic pulmonary artery pressure is significantly correlated with poorer prognosis, though symptomatic improvement post-TTVI remained achievable regardless of PH severity.^24^

Contemporary outcomes from European post market registries (bRIGHT, PASTE, and TRICLASP) affirm the effectiveness and safety of transcatheter interventions. The bRIGHT TriClip registry reported excellent device success rates and symptomatic improvement, highlighting anatomical predictors for successful outcomes such as reduced leaflet tethering and smaller right atrial volumes.^9^ Recent European registry findings strongly support early and precise risk stratification using TRISCORE and TRIVALVE scores, supplemented by clinical markers including right heart failure symptoms, nutritional status, RV function indices, and PH presence. Early intervention, guided by comprehensive risk assessment, offers significant prognostic and symptomatic benefits, reshaping TR management paradigms. Continued integration of these markers and scores into clinical practice will optimize timing and selection of surgical or transcatheter interventions, enhancing patient outcomes significantly.

### Outcome of T-TEER procedures

Recognizing its efficacy and safety, European registries soon focused on T-TEER. An acute reduction to a residual TR ≤2/5 can be expected in up to 77–87%, with a median reduction by two grades.^15,25^ Short-term durability proved to be promising, with good results in 72–83% at 12 months. Clinically, T-TEER led to an improvement of NYHA functional class by a median of two grades, increasing the proportion of patients graded as NYHA I/II to 55–69%. All-cause mortality after 1 year was <20%.^26^

Similar results were achieved in prospective studies. Acutely, the reduction to TR ≤ 2/5 was 77–90% and sustained in 81% at one year.^12,14^ Aside increasing the functional capacity a rise in 6-min walking distance by 38.2 m and in the Kansas City Cardiomyopathy Questionnaire Score by 13–19 points was achieved as a short-term result. These outcomes were stable after 12 months, with 75% being in NYHA functional class I/II, and a further increase in the Kansas City Cardiomyopathy Questionnaire Score by 19 ± 26 points. All-cause mortality at 30 days ranged from 1.0% to 1.5%, and at 1 year, it was 15.1% mainly as a result of the concomitant morbidity of this patient population.^14^

## Mechanistic effects

In response to leaflet approximation, a reduced valve area and increased transvalvular gradients are to be expected using T-TEER. Balancing effectively reducing TR and avoiding tricuspid valve stenosis, evidence on the clinical impact of postinterventional mean tricuspid valve gradient is of great interest for operators. A retrospective analysis of the TriValve registry investigated associations of mean gradient with clinical outcomes (all-cause mortality and heart failure hospitalization).^27^ Baseline mean gradient and the number of implanted devices were associated with a higher postinterventional mean gradient. Importantly, an increase in tricuspid valve gradients was not associated with adverse outcomes following TTVI.

Successful TTVI has also been associated with improved left ventricular filling and forward flow towards the pulmonary artery and reduced RV preload.^28^ The TriValve registry further evaluated several postinterventional mechanistic effects in patients undergoing TTVI, one of it being the evolution of concurrent mitral regurgitation severity.^29^ Physiologically, one could expect MR severity to increase after TTVI, in response to preload augmentation. However, there were no real-world data on that topic yet. Patients with prior mitral valve interventions were excluded, so that a TriValve subgroup analyses included 359 patients, of which 80% were treated with T-TEER. Interestingly, MR improvement was found in about a third of patients at discharge and 2-month follow-up, while in only about 10% of patients, mitral regurgitation worsening was observed. Annuloplasty and heterotopic tricuspid valve replacement were associated with mitral regurgitation worsening, while amongst others T-TEER, acute procedural success and TR reduction were associated with mitral regurgitation improvement. Mitral regurgitation evolution was not associated with patient survival.

Haemodynamically, patients undergoing TTVI might be at risk of postinterventional low-cardiac output syndrome due to increased RV afterload (afterload mismatch). A substudy of the TriValve registry investigated the postinterventional occurrence of low-cardiac output defined as hypotension necessitating inotropic support.^30^ Of 500 patients included in this analysis, only 2.8% (*n* = 14) suffered from low-cardiac output after TTVI, that was associated with higher in-hospital mortality (43% vs. 1%). Significant predictors were baseline biventricular impairment, procedural complications, preprocedural haemodynamic instability and EuroScore >15%. As far as this may be accurately assessed retrospectively, acute RV dysfunction was the underlying reason for low-cardiac output in only 2 of 14 patients, both of which recovered quickly and had a further uneventful hospital stay. Overall, this analysis observed very low rates of acute RV dysfunction leading to postinterventional low-cardiac output, supporting the safety of TTVI.

## Transcatheter tricuspid valve replacement (TTVR) as alternative to T-TEER

In patients unsuitable for T-TEER, TTVR was sought to successfully address most severe TR. While early designs ultimately had to be abandoned, favourable results were achieved in a multicentre first-in-human experience in 2021, demonstrating early effectiveness

While TTVR achieves more complete TR elimination, it carries a different risk profile.^31^ T-TEER showed low 30-day mortality (1.1%) in TRILUMINATE, with 1-year mortality at 9.4%.^25,32^

TTVR trials report similar 1-year mortality rates (∼15%), although with higher rates of complications: 30-day mortality ranged from 2.2% to 8%, with major bleeding in 15–18% and new pacemaker implantation in up to 15.8%.^33,34^ These factors highlight the importance of matching therapy choice to individual risk and anatomy.

## Procedural planning and patient selection for T-TEER vs. TTVR

**Figure 1.**
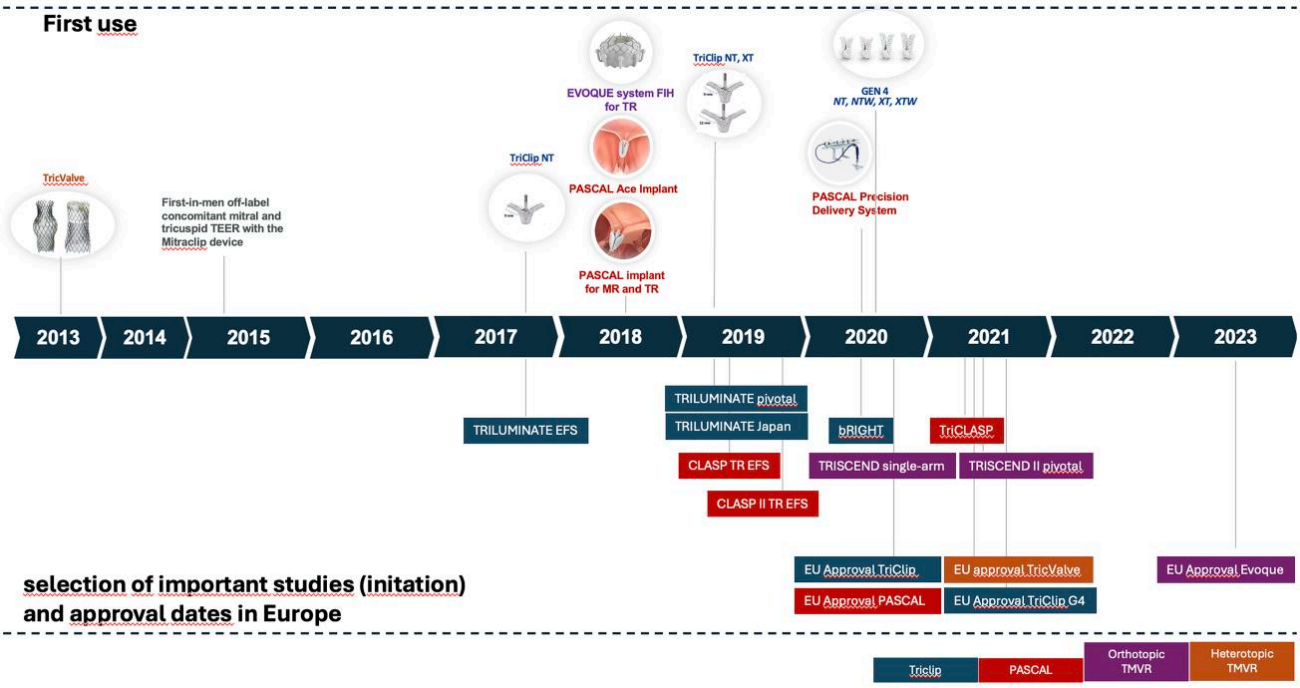
First use, selection of early feasibility, and pivotal of currently approved devices for the treatment of tricuspid regurgitation. EFS, early feasibility study, TR, tricuspid regurgitation.

**Figure 2.**
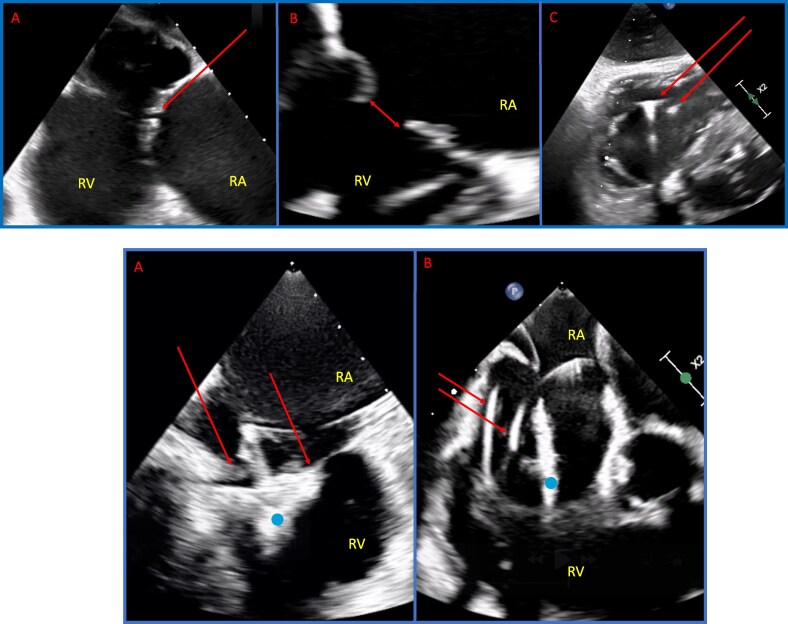
Anatomical and imaging considerations for transcatheter tricuspid valve interventions. Upper left: Transesophageal images showing adequate image quality with desirable anatomical features for T-TEER with good leaflet length, mobile leaflets, and a reasonable coaptation gap (red arrow); Upper middle: Transesophageal echocardiographic images showing sub-optimal anatomical features for tricuspid TTEER, with a wide coaptation gap (red arrow). This patient was referred for TTVR; Upper right: Transesophageal echocardiographic images showing sub-optimal image caused by artefact from two transvalvular pacing leads (red arrows). This patient underwent T-TEER using ICE, see lower panel. ICE images demonstratings the advantage of intracardiac imaging in challenging anatomies. Lower left: Clear images of the clip (blue dot) and valve leaflets (red arrows) during T-TEER, despite the presence of pacing leads, in the patient from Figure 1C. Lower right: Images of pacing leads (red arrows) and clip/delivery (blue dot) catheter in the same patient. ICE, intracardiac echocardiography; RA, right atrium; RV, right ventricle; T-TEER, transcatheter tricuspid valve edge-to-edge repair; TTVR, transcatheter tricuspid valve replacement; TV, tricuspid valve.

**Figure 3.**
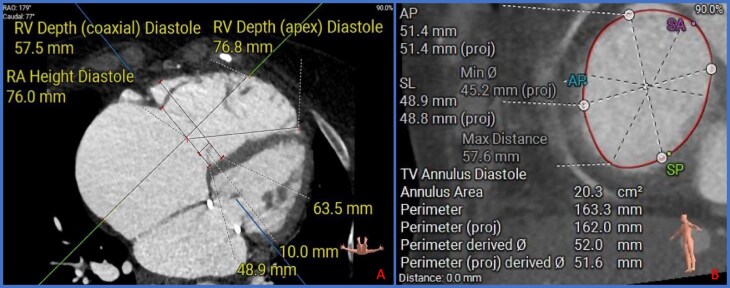
CT scan sections from a TTVR planning report illustrating dimensions for TTVR planning; (A) Annular dimensions in the diastolic phase. (B) Four-chamber view in the diastolic phase showing ventricular and atrial dimensions and depths for procedural planning. CT, Computed tomography; TTVR, transcatheter tricuspid valve replacement.

Multi-modality imaging is central to procedural planning. Transthoracic and transoesophageal echocardiography (TTE) and (TEE) assess valve structure, lesion severity, and chamber function.^35^ For TTVR, cardiac computed tomography (CT) is vital for sizing and assessing anatomic relationships.^36^ CT and TEE must be performed under optimized, euvolemic conditions to prevent errors in measurement. Intracardiac echocardiography (ICE) is an alternative in patients with contraindications to TEE or poor image quality. However, owning to regulations and reimbursement availability, ICE is currently less widely available Europe than in the USA.^37,38^ A summary of imaging considerations is included in *Figures 1–3*.

Right heart catheterization helps to determine pulmonary pressures and RV–PA coupling, which guide risk stratification and predict RV adaptation post-procedure.^10,36,39^ In patients with pre-capillary PH or RV–PA uncoupling, TTVR may carry excessive risk, while T-TEER remains feasible in select cases.^40^

Ideal T-TEER candidates have symptomatic TR, preserved RV function, and favourable anatomy (e.g. coaptation gap ≤7 mm, mobile leaflets).^10^ TTVR eligibility (per TRISCEND-II) excludes those with severe PH or advanced ventricular dysfunction.^41^ Risk scores such as TRI-SCORE and TRIVALVE offer additional stratification.^15^ These tools are increasingly used in European centres, promoted by European guideline-directed heart team evaluation.^42^

T-TEER success is linked to leaflet mobility, coaptation gap, and sub-valvular anatomy. The GLIDE score uses echocardiographic features to predict feasibility.^43^ More relevant to TTVR are annular dimensions and device interactions with coronary structures and transvalvular leads must also be considered. While GLIDE scores of 4 or 5 points might be considered as thresholds for the consideration of TTVR, the optimal interventional therapy for patients with GLIDE scores of 2 or 3 points are less clear.^36^

Pre-existing pacemaker leads may complicate both, T-TEER and TTVR due to anchoring interference or lead impingement.^41^ Pre-existing conduction abnormalities have been associated with an increased risk for pacemaker need after TTVR. Leadless pacing, coronary sinus lead pacing or epicardial systems may be alternatives when traditional pacing is not feasible after TTVR, however pre-procedural collaboration with electrophysiologists is critical.^25^

## Anticoagulation and bleeding risk

TR patients have a high baseline risk of bleeding due to congestion-related organ dysfunction.^2^ TTVR requires long-term anticoagulation to prevent prosthetic thrombosis and consequently major bleeding is more common post-TTVR (up to 27%) than post-T-TEER (4.7–11.9%).^10,36^

### Summary

Interventional treatment of TR has evolved within a few years to an established method. This fast development was accompanied by a robust data acquisition in both controlled trials and large-scale registries. The European experience is documented in numerous of these publications and paved the way towards the upgraded recommendation in the latest ESC guidelines.

## Data Availability

No new data were generated or analysed in support of this research.
